# Early Serum Infliximab Levels in Pediatric Ulcerative Colitis

**DOI:** 10.3389/fped.2021.668978

**Published:** 2021-07-29

**Authors:** Jennifer C. C. deBruyn, Kevan Jacobson, Wael El-Matary, Eytan Wine, Matthew W. Carroll, Caitlin Goedhart, Remo Panaccione, Iwona T. Wrobel, Hien Q. Huynh

**Affiliations:** ^1^Department of Pediatrics, University of Calgary, Calgary, AB, Canada; ^2^Department of Community Health Sciences, University of Calgary, Calgary, AB, Canada; ^3^Department of Pediatrics, University of British Columbia, Vancouver, BC, Canada; ^4^Department of Pediatrics, University of Manitoba, Winnipeg, MB, Canada; ^5^Department of Pediatrics, University of Alberta, Edmonton, AB, Canada; ^6^Department of Medicine, University of Calgary, Calgary, AB, Canada

**Keywords:** infliximab, ulcerative colitis, pediatrics, induction, serum concentration

## Abstract

**Background:** Data on serum infliximab concentrations during induction in pediatric ulcerative colitis are limited. The study aim is to evaluate the relationship between serum infliximab concentrations during induction and short-term clinical remission in children with ulcerative colitis.

**Methods:** We carried out a prospective, multi-center cohort study in pediatric patients with ulcerative colitis. Serum infliximab concentrations were collected at peak dose #1, week 1, trough pre-dose #2, and trough pre-dose #3. Infliximab dosing was left to investigator discretion. Clinical remission was defined by pediatric ulcerative colitis activity index <10 at week 8.

**Results:** Twenty-four of thirty-four subjects (71%) achieved clinical remission at week 8. The median infliximab concentrations were 33.0 μg/mL (interquartile range: 26.5–52.1 μg/mL) pre-dose #2 and 22.5 μg/mL (interquartile range:15.9–32.3 μg/mL) pre-dose #3. Trough pre-dose #2 infliximab concentration yielded area under receiver operator characteristic curve 0.7, 95% CI: 0.5–0.9 in predicting week 8 clinical remission; a cut-off of 33.0 μg/mL yielded 62.5% sensitivity, 66.7% specificity. Trough pre-dose #3 infliximab concentrations were lower for subjects <10 years compared to ≥ 10 years [median 15.9 μg/mL, interquartile range (IQR) 8.5–21.8 μg/mL vs. 27.7 μg/mL, IQR 17.2–46.7 μg/mL, *p* = 0.01] and correlated with baseline weight (Spearman's rank correlation coefficient 0.45, *p* = 0.01). The median half-life following first IFX dose was 6.04 days (IQR 5.3–7.9 days).

**Conclusions:** Infliximab concentrations ≥33 μg/mL prior to the second dose were associated with week 8 clinical remission. As young age and low body weight impact infliximab concentration, prospective studies with proactive adjustment in pediatric patients with ulcerative colitis should be carried out. Clinicians caring for children with UC should diligently adjust and monitor infliximab to optimize response.

## Introduction

Infliximab (IFX) is a monoclonal antibody against tumor necrosis factor α (TNF-α) used in adult and pediatric ulcerative colitis (UC) that is initiated in up to 31% of newly diagnosed children with UC by 52 weeks of follow-up (1–5). However, in children with moderate to severely active UC who commence IFX, 26.7% fail to respond to induction and up to 39% have inadequate initial response or lose response and require colectomy by 24 months (2, 6). Therefore, strategies to optimize IFX response are still desired.

Numerous factors affect pharmacokinetics of IFX. In adults with UC, higher body weight, lower serum albumin, antibodies against IFX (ATI), and female sex accelerated clearance (7). In children with UC who may have differing weights and disease burden compared to adults, limited data are available. In a single study, age and baseline use of concomitant immunomodulators did not influence IFX pharmacokinetics and the effects of weight, serum albumin, ATI, and sex were not evaluated (8). A recent study of children with IBD suggested that young age may further affect IFX pharmacokinetics; the majority of young children (age <10 years) had inadequate IFX trough levels at the start of maintenance and were more likely to have an intensive treatment regimen for the first year of IFX treatment compared to older children (9).

Serum IFX concentrations correlate with clinical and endoscopic outcomes in UC (10). Limited studies have evaluated IFX levels during induction of active UC, a distinctive disease state with high inflammatory burden. In adults with UC, IFX concentration thresholds during induction are associated with endoscopic response and short-term mucosal healing (11, 12). In children with UC, there are limited data on IFX concentrations during induction. In this study, we evaluated the relationship between serum IFX concentrations during induction and short-term clinical remission in children with UC. We also examined potential factors associated with IFX concentrations, pharmacokinetics, and achievement of short-term clinical remission.

## Methods

### Study Design and Conduct

This multicenter prospective observational cohort study was conducted at four Western Canadian pediatric tertiary-care hospitals (British Columbia Children's Hospital, Vancouver; Alberta Children's Hospital, Calgary; Stollery Children's Hospital, Edmonton; and Children's Hospital, Winnipeg). The inclusion criteria were: diagnosis of UC (13, 14), plan to start IFX, age ≤ 18 years, and negative stool culture and *Clostridium difficile* toxin assay. The exclusion criteria were: concomitant known GI disorder, intravenous immunoglobulin administration, and prior anti-TNF medications. Concomitant therapy with 5-aminosalicylates (5-ASA), azathioprine, 6-mercaptopurine, methotrexate, corticosteroids, and antibiotics were permitted. Subjects were enrolled between November 20, 2013 and August 22, 2016.

Baseline data were collected at week 0 on demographics, medical history, anthropometry, disease classification (15), and clinical activity [Pediatric Ulcerative Colitis Activity Index (PUCAI) (16)]. We collected serum for hemoglobin, albumin, erythrocyte sedimentation rate [ESR], c-reactive protein [CRP], platelet count and stool for fecal calprotectin. We used the BÜHLMANN Calprotectin ELISA test kit (ALPCO Diagnostics, NH, USA). Follow-up data collected at weeks 1, 2, and 6 included PUCAI score, anthropometry, medication history, and laboratory tests.

Subjects received IFX (Remicade^®^ Janssen Biotech, Inc., Horsham, Pennsylvania, USA) with a typical induction of IFX 5 mg/kg at weeks 0, 2, and 6; this could be adjusted at clinician's discretion. All doses and dates were recorded. Intensified IFX schedule was defined as IFX dose #3 ≥ 7 mg/kg and/or time from dose #1 to #3 ≤ 35 days. This stringent definition acknowledged practice patterns of rounding up IFX dose to nearest hundred and accounted for scheduling issues.

### Serum Infliximab and Antibody to Infliximab Concentrations

Serum IFX and ATI concentrations were collected at peak dose #1 (1-hour post-infusion), week 1, trough pre-dose #2, and trough pre-dose #3. Sera were processed at Prometheus Laboratories Inc., San Diego, California using the drug-tolerant PROMETHEUS^®^ Anser^®^ IFX liquid-phase mobility shift assay. The lower limit of detection was 0.98 μg/mL for serum IFX and 6.6 U/mL for ATI with no upper limit of quantification. Physicians were blinded to results.

### Outcome

At week 8, subjects underwent PUCAI assessment. The primary outcome was clinical remission (PUCAI <10 at week 8, in absence of colectomy) (16). A secondary outcome was clinical response at week 8 (at least 50% reduction in PUCAI from baseline).

### Statistical Analysis

Data are presented as medians (interquartile range [IQR]). Categorical data were compared using χ^2^ or *Fisher's* exact test and continuous data using the *Student t*-test, *Wilcoxon* rank sum test, or *Wilcoxon* signed-rank test, as appropriate.

Receiver operating characteristic (ROC) curves were used to assess the diagnostic ability of models of serum IFX concentrations at week 1, pre-dose #2, and pre-dose #3 in predicting clinical remission at week 8. Area under ROC (AUROC) values were interpreted as AUC <0.70 corresponding to low diagnostic accuracy; 0.70–0.90 = moderate diagnostic accuracy; and AUC ≥ 0.90 = high diagnostic accuracy. The clinical utility of a range of pre-dose #2 and pre-dose #3 IFX levels are shown reporting sensitivity, specificity, positive predictive value, and negative predictive value. The Youden Index was used to select an optimal threshold IFX concentration in models that showed moderate diagnostic ability. ROC curves were also used to assess the diagnostic ability of models of serum IFX concentrations at week 1, pre-dose #2, and pre-dose #3 in predicting CRP remission (<10.0 mg/L) and ESR remission (<10.0 mm/h) week 8.

Univariate logistic regression analysis was performed to evaluate association of clinical and laboratory variables with week 8 clinical remission. The variables evaluated included: male gender, young age (<10 years, based on Paris Classification) (15), high CRP (>10.0 mg/L), high ESR (>10.0 mm/h), low albumin (<33 g/L), concomitant immunomodulator, body mass index (BMI), and intensified IFX schedule. This analysis was repeated for outcome of steroid-free clinical remission, and achievement of clinical response and/or remission. The CRP, ESR, and albumin interpretation were based on the upper and lower limits of normal from participating centers' laboratories.

If an AUROC demonstrated moderate diagnostic ability, then univariate logistic regression analysis was performed to evaluate association between the above variables and achievement of the cut-off serum IFX concentration, according to the above analysis.

The Kruskal-Wallis test was performed to determine if there were statistically significant differences between serum IFX concentrations pre-dose #2 among categories differentiated by: male gender, young age (<10 years, based on Paris Classification) (14), high CRP (>10.0 mg/L), high ESR (>10.0 mm/h), low albumin (<33 g/L), concomitant immunomodulator, body mass index (BMI), intensified IFX schedule, and IFX dose #1 ≥ 7 mg/kg. A similar analysis was performed for serum IFX concentrations pre-dose #3 using the above variables with the addition of dose #3 ≤ 35 days from dose #1. By determining *Spearman'*s rank correlation coefficient, we evaluated for association between serum pre-dose #2 and pre-dose #3 IFX concentrations and age, baseline laboratory parameters (CRP, ESR, albumin), body mass index, and baseline infliximab dose (mg/kg).

We calculated the half-life associated for dose #1. We compared IFX dose, regimen, concentrations, and half-life by age [categorical variable (young age <10 years) (15), *Wilcoxon* rank-sum test] and by anthropometric measures and age [continuous variables, *Spearman'*s rank correlation coefficient].

Results were considered statistically significant when *P* < 0.05.

We aimed to enroll 32 children for this pilot study to evaluate early induction IFX concentrations to guide future prospective studies implementing strategies to optimize IFX response in pediatric UC. Subjects lost to follow-up were excluded from overall analysis.

Data were entered into Research Electronic Data Capture (REDCap). Statistical analysis was performed using Intercooled Stata 13 software (Stata Corporation, College Station, Texas).

### Ethics

This study was approved by the institutional review board at each institution. All participants provided written consent.

## Results

Thirty-five children consented; one subject was lost to follow-up after dose #1; therefore 34 subjects completed follow-up and are included in this analysis. Baseline characteristics are presented in Table 1. At baseline, 14 (41.2%) subjects had elevated CRP (≥10 mg/L), 33 (97.1%) had high ESR (>10.0 mm/h), 11 (32.4%) had low albumin, and eight (23.5%) were <10 years of age.

**Table 1:** Baseline characteristics.

**Characteristic**	
**Age at diagnosis, years**	
Median (IQR)	12.5 (8.0–14.2)
**Age at enrolment, years**	
Median (IQR)	13.3 (10.4–14.9)
**Time from diagnosis to enrolment, years**	
Median (IQR)	0.8 (0.2–1.9)
**Sex**, ***n*****(%)**	
Male	13 (38.2)
**Indication**, ***n*****(%)**	
Acute severe	18 (52.9)
Chronic active	12 (35.3)
Other	4 (11.8)
**Baseline pediatric ulcerative colitis activity index**	
Median (IQR)	47.5 (30–70)
**Paris classification**, ***n*****(%)**	
Ulcerative proctitis	1 (2.9)
Left sided ulcerative colitis	4 (11.8)
Extensive ulcerative colitis	7 (20.6)
Pancolitis	19 (55.9)
Unknown	3 (8.8)
**Medication**, ***n*****(%)**	
**5-Aminosalicylates–oral**	
Past	9 (25.7)
Current	19 (55.9)
Stopped during infliximab induction	7 (36.8)
**Corticosteroids–oral**	
Past	8 (23.5)
Current	27 (79.4)
Stopped during infliximab induction	14 (51.9)
**Corticosteroids**–**Intravenous**	
Past	7 (20.6)
Current	3 (8.8)
Stopped during infliximab induction	3 (100.0)
**Azathioprine**	
Past	3 (8.8)
Current	20 (58.8)
Stopped during infliximab induction	2 (10.0)
**Methotrexate**	
Past	0
Current	10 (29.4)
Stopped during infliximab induction	1 (9.1)

*IQR, interquartile range*.

The IFX induction regimen is presented in Table 2 and Supplementary Figure 1. Eleven subjects started with dose #1 ≥ 7 mg/kg (32.4%). During induction, five subjects had dose escalation for breakthrough symptoms (two at dose # 2; three at dose #3) and five subjects had frequency shortening (one received dose #2 at <12 days from dose #1; four received dose #3 at < 21 days from dose #2). In total, 13 subject were classified as intensified IFX induction (nine based on IFX dose #3 ≥ 7 mg/kg alone, one based on time from dose #1 to #3 ≤ 35 days alone, and three based on both dose and time).

**Table 2:** Infliximab schedule and serum infliximab concentration.

	**Infliximab dose (mg/kg)**	**Time from baseline to infliximab level (days)**	**Serum infliximab concentration (μg/mL)**
Dose #1, median (IQR)	6.2 (5.6–8.1)	–	Peak 186.1 (150.8–227.5)^a^
Week 1, median (IQR)	–	7 (7, 8)	60.4 (51.0–81.8)^b^
Dose #2, median (IQR)	6.0 (5.4–8.2)	14 (13, 14)	Trough 33.0 (26.5–52.1)^c^
Dose #3, median (IQR)	6.0 (5.5–8.0)	42 (41.5–43.0)	Trough 21.8 (15.9–32.3)^d^

*Data available for*

a *n = 28 subjects*,

b *n = 23 subjects*,

c *n = 32 subjects*,

d *n = 31 subjects*.

*IQR, interquartile range*.

At week 8, twenty-four subjects (70.6%) achieved clinical remission, including 16 who were not on corticosteroids (47.0%); 28 subjects (82.4%) achieved clinical response. One subject underwent colectomy after 2 doses of IFX (at 32 days from dose #1) and did not receive any further IFX. Excluding the subject with colectomy, the median PUCAI score at week 8 was 0 (IQR: 0–15). At week 8, the PUCAI score significantly decreased compared to baseline (*p* < 0.0001). ESR, CRP, hemoglobin, platelets, and albumin showed significant improvement from baseline (Table 3). Of 14 subjects with elevated baseline CRP, 12 were considered in CRP remission at week 6; in total, 31 (91.2%) had normal CRP at week 6. Fourteen (41.2%) were considered in ESR remission at week 6; all 14 had elevated baseline ESR. The median fecal calprotectin levels were 2,057 mcg/g (IQR 1,081–5,307, *n* = 13) at week 0, 1,110 mcg/g (IQR 199–3,321, *n* = 13) at week 2, and 1,552 mcg/g (IQR 1,391–3,254, *n* = 17) at week 6. Of the subjects who provided week 6 fecal calprotectin, 3 were considered in clinical remission with fecal calprotectin <200 mcg/g; the remaining 14 subjects had fecal calprotectin levels >800 mcg/g (with 9 of 14 subjects considered in clinical remission).

**Table 3:** Key laboratory results.

**Test [Median (IQR)]**	**Baseline**	**Pre-dose #3**	***P*-value**
ESR (mm/h)	22 (17–34)	12 (7–21)	0.0003
CRP (mg/L)	5.0 (2.8–21.0)	1.3 (1–5)	0.008
Hemoglobin (g/L)	102 (85–116)	117 (104–125)	0.0007
Platelets (×10^9^/L)	461 (355–539)	345 (296–433)	0.0002
Albumin (g/L)	34 (31–40)	40 (36–44)	0.005

*ESR, erythrocyte sedimentation rate; CRP, c-reactive protein*.

Thirty subjects were on corticosteroids at baseline. Two subjects started or increased prednisone during the study period. At week 8, ten patients remained were on corticosteroids, of which nine were on a weaning regimen with dose ≤ 20 mg/d; eight of these nine patients on weaning corticosteroids were in clinical remission.

### IFX Concentrations and Week 8 Outcomes

**Figure 1:**
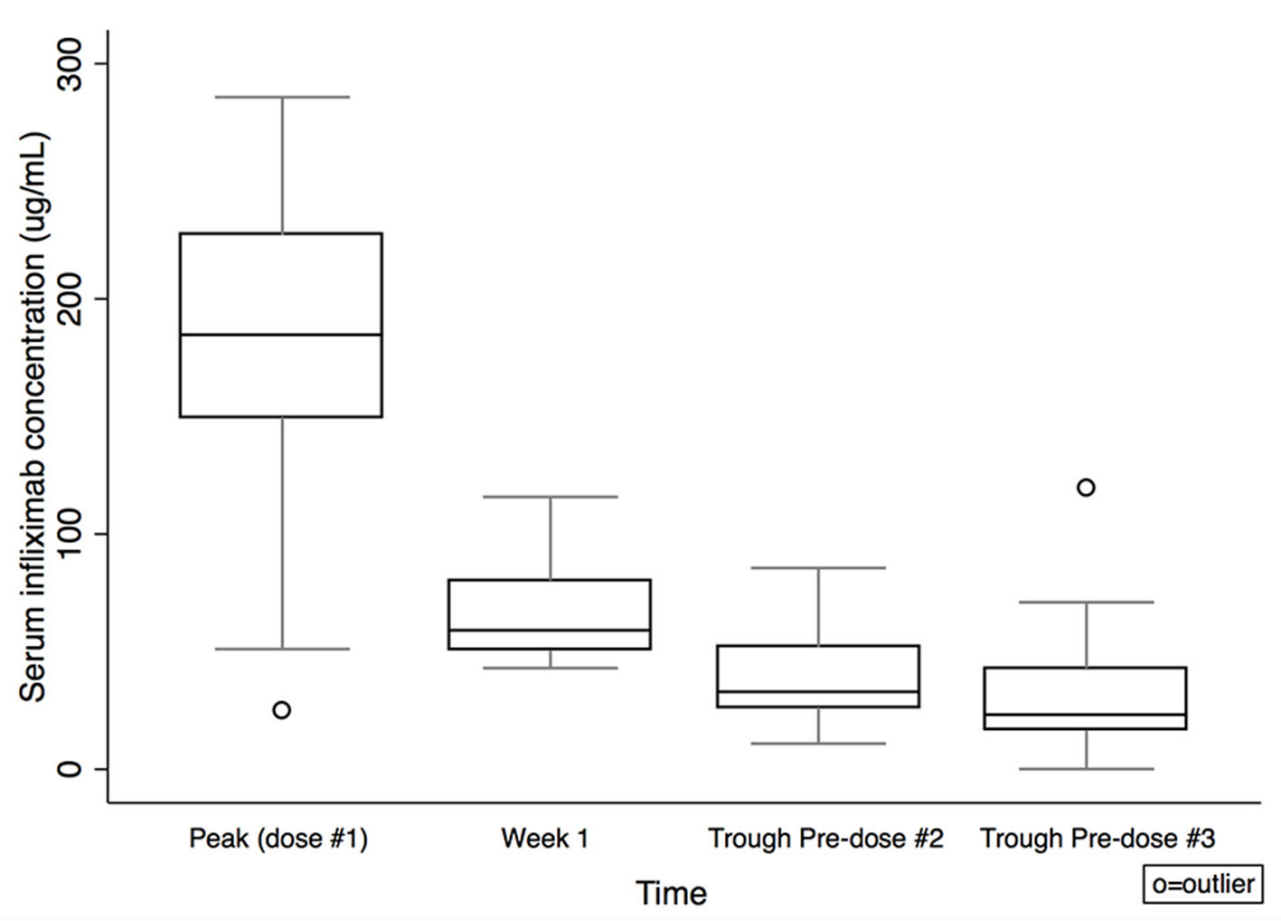
Serum infliximab concentrations by time. Box plot values include: lower adjacent value, first quartile, median, third quartile, and upper adjacent value (in ascending order). Lower and upper adjacent values are the most extreme values within the first quartile minus 1.5 × interquartile range and the third quartile plus 1.5 × interquartile range, respectively.

**Figure 2:**
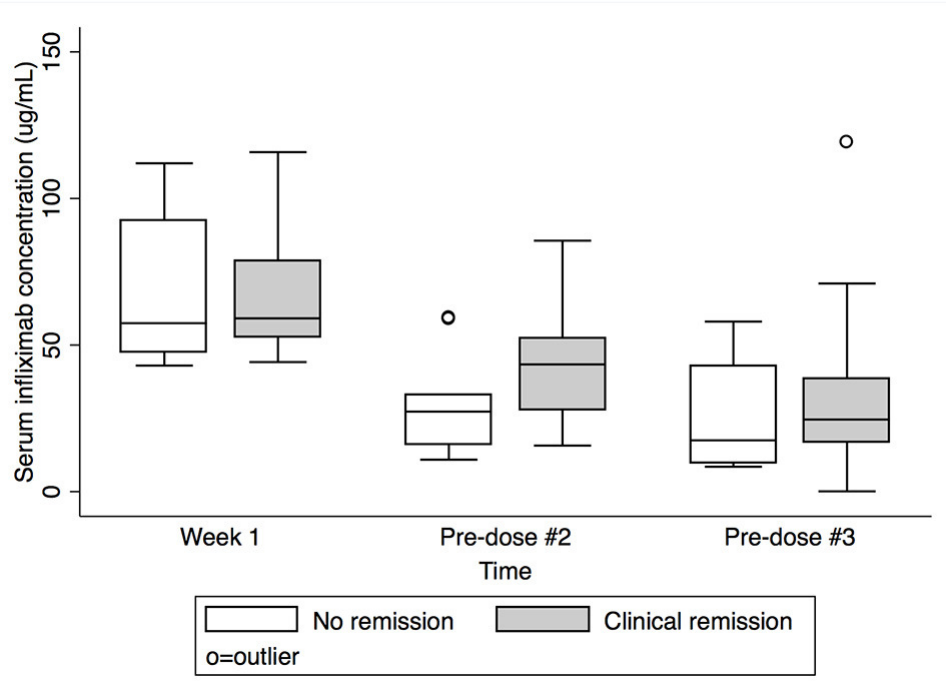
Serum infliximab concentrations at week 1, pre-dose #2, and pre-dose #3 by clinical remission status at Week 8. Box plot values include: lower adjacent value, first quartile, median, third quartile, and upper adjacent value (in ascending order). Lower and upper adjacent values are the most extreme values within the first quartile minus 1.5 × interquartile range and the third quartile plus 1.5 × interquartile range, respectively.

Serum IFX concentrations are shown in Table 2 and Figure 1. Comparing subjects in clinical remission at week 8 with non-remitters, there was no statistically significant difference in IFX concentrations at peak or week 1. There was a trend for higher IFX concentrations at pre-dose #2 and #3 for those in clinical remission at week 8 vs. non-remitters (pre-dose #2 median: 43.4 μg/mL, IQR 27.8–52.3 vs. 27.3 μg/mL, IQR 16.2–32.9; *p* = 0.08; pre-dose #3: 24.6 μg/mL, IQR 16.9–38.6 vs. 17.5 μg/mL, IQR 9.7–42.9; *p* = 0.4; Figure 2). There was a trend, but no statistically significant difference in IFX concentrations at pre-dose #2 and #3 for those in clinical response and/or remission at week 8 vs. non-responders/remitters (pre-dose #2 median: 42.2 μg/mL, IQR 27.1–52.3 vs. 30.4 μg/mL, IQR 19.3–32.9; *p* = 0.4; pre-dose #3: 24.6 μg/mL, IQR 16.9–44.8 μg/mL vs. 17.5 μg/mL, IQR 9.7–23.7; *p* = 0.3)

The AUROC yielded moderate diagnostic ability for predicting week 8 clinical remission using the pre-dose #2 IFX concentration (AUROC 0.70, 95% CI 0.47–0.93) (Supplementary Figure 1). The AUROC models yielded low diagnostic ability for predicting week 8 clinical remission using IFX concentration at week 1 (AUROC 0.55, 95% CI 0.19–0.94) and pre-dose #3 (AUROC 0.61, 95% CI 0.34–0.89) (Supplementary Figure 1). The sensitivity, specificity, positive predictive value, and negative predictive value for a range of serum IFX concentrations at pre-dose #2 and pre-dose #3 are shown in Supplementary Table 1. As the AUROC model for IFX concentration pre-dose #2 showed moderate diagnostic ability, we used Youden Index to identify a pre-dose #2 cutoff of 33.0 μg/mL which yielded sensitivity 62.5%, specificity 66.7%, PPV 83.3%, NPV 40.0%.

Serum IFX concentrations at week 1, pre-dose #2, and pre-dose #3 did not differ by week 6 CRP or ESR remission status, though there may be a trend for higher pre-dose #2 IFX concentrations for week 6 CRP remission *vs* non-remitters (pre-dose #2 median: 33.0 μg/mL, IQR 27.3–52.3 μg/mL vs. 16.2 μg/mL, IQR 10.9–43.4; *p* = 0.099).

IFX pre-dose #2 and pre-dose #3 concentrations were not associated with male gender, baseline CRP, ESR, albumin, IFX dose #1 ≥ 7 mg/kg, BMI, or intensified IFX schedule (evaluated for pre-dose #3 only). Concomitant immunomodulator was not evaluated as only 4 subjects were not on concomitant immunomodulator.

### Anti-drug Antibodies

One subject who was on concomitant azathioprine during the entire study period developed ATI (9.2 U/mL) with undetectable serum IFX concentrations (<1.0 μg/mL) at pre-dose #3. This subject developed clinical symptoms prior to dose #3, received systemic corticosteroids and dose #3 escalation, and ultimately achieved clinical remission by week 8.

### Evaluation of Predictors of Week 8 Outcomes

In a univariate analysis, achievement of clinical remission at week 8 was not associated with male gender, young age, high baseline CRP, low baseline albumin, BMI, or intensified IFX schedule (Supplementary Table 2). High ESR was not evaluated as only one subject did not have high ESR at baseline. Concomitant immunomodulator was not evaluated as only 4 subjects were not on concomitant immunomodulator. Similarly, no variables of association were identified using the outcome of corticosteroid-free clinical remission, clinical response and/or remission, CRP remission, nor ESR remission.

### Association of Young Age and Low Baseline Weight With Lower IFX Concentration at Week 6

**Figure 3:**
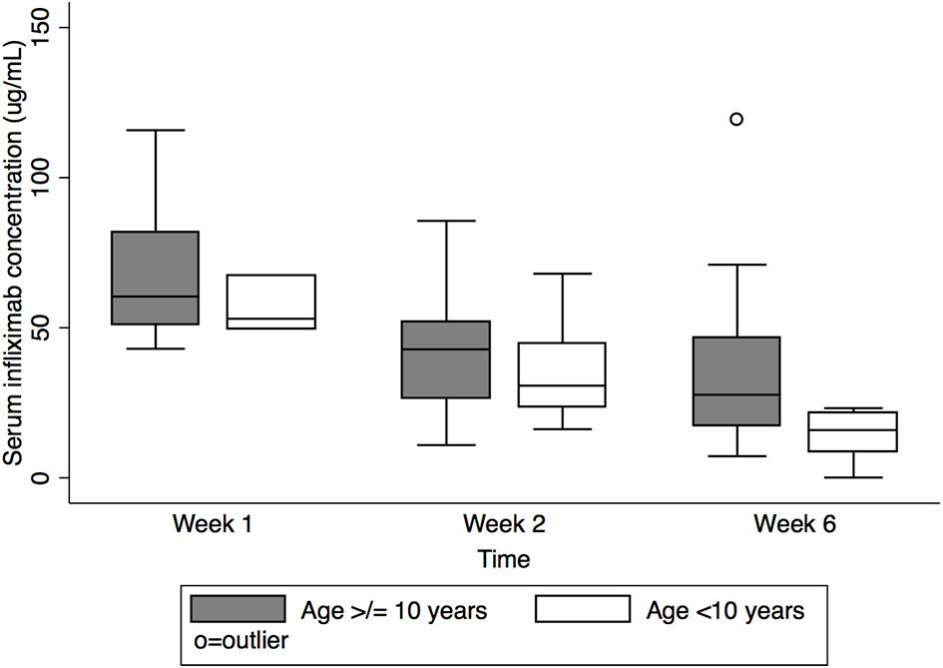
Serum infliximab concentrations at week 1, pre-dose #2, and pre-dose #3 by age. Box plot values include: lower adjacent value, first quartile, median, third quartile, and upper adjacent value (in ascending order). Lower and upper adjacent values are the most extreme values within the first quartile minus 1.5 × interquartile range and the third quartile plus 1.5 × interquartile range, respectively.

Pre-dose #3 IFX concentration differed by age (Figure 3) [median 15.9 μg/mL, IQR 8.5–21.8 μg/mL age <10 years vs. 27.7 μg/mL, IQR 17.2–46.7 μg/mL age ≥ 10 years (*p* = 0.01)]. Though there was a similar trend at week 1 and pre-dose #2, this did not reach statistical significance (median week 1: 53.0 μg/mL, IQR 49.5–67.5 μg/mL for age <10 years vs. 60.4 μg/mL IQR 51.0–81.8 μg/m L for age ≥10 years, *p* = 0.5; pre-dose #2: 30.7 μg/mL, IQR 23.6–44.6 μg/mL for age <10 years vs. 42.9 μg/mL, IQR 26.5–52.1 μg/mL for age ≥10 years, *p* = 0.6).

**Figure 4:**
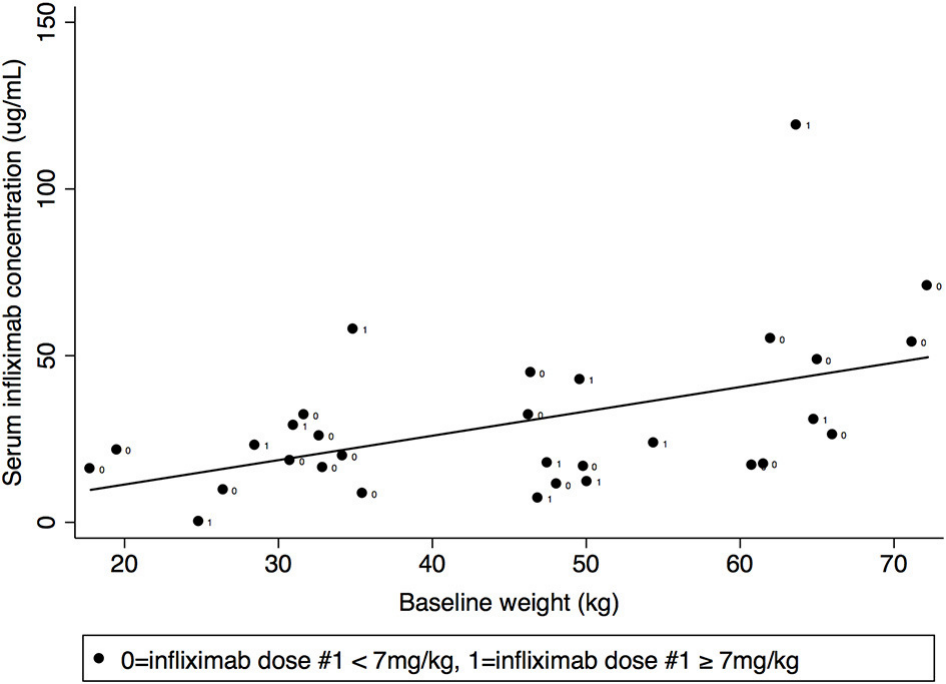
Pre-dose #3 trough serum infliximab concentration by baseline subject weight.

Pre-dose #3 IFX concentration correlated with baseline weight (Spearman's rank correlation coefficient 0.45, *p* = 0.01) but did not correlate with baseline BMI (Figure 4). IFX concentrations at week 1 and pre-dose #2 did not correlate with baseline weight or BMI.

IFX dose #1 did not differ by age (median 5.6 mg/kg, IQR 5.0–6.8 mg/kg for age <10 years vs. 6.4 mg/kg, IQR 5.8–8.4 mg/kg for age ≥10 years, *p* = 0.1) and did not correlate with baseline weight or BMI. IFX intensification was not associated young age (<10 years) (OR 0.14, 95% CI 0.003–1.45) and did not correlate with baseline weight.

### Infliximab Half-Life

Twenty subjects provided sufficient measurements to calculate IFX dose #1 half-life. The median half-life was 6.04 days (IQR 5.3–7.9 days); there was no difference between subjects in clinical remission at week 8 (median 6.68 days, IQR 5.35–9.03 days) vs. not in clinical remission (5.50 days, IQR 4.97–5.92 days) (*p* = 0.2). IFX half-life did not correlate with age; however only three of these 20 subjects were <10 years of age. IFX half-life did not correlate with baseline weight, baseline BMI, or dose #1 amount (mg/kg).

## Discussion

In this prospective multicenter cohort study of children with UC, we evaluated early IFX concentrations during IFX induction. Our results demonstrate that the majority (70%) of children with UC who undergo IFX induction achieve clinical remission by week 8. We demonstrate that pre-dose #2 IFX concentrations have moderate diagnostic ability to predict week 8 clinical clinical remission, with an IFX concentration ≥33.0 μg/mL yielding best test characteristics, including PPV 83%. Though there was a trend toward higher pre-dose #2 and pre-dose #3 serum IFX concentrations for subjects in clinical remission at week 8 compared to subjects not in clinical remission, this did not reach statistical significance, potentially due to larger required sample size to demonstrate this difference.

IFX induction concentrations have been described in adults with UC. Papamichael et al. demonstrated in a retrospective, single-center study of adults with UC similar serum IFX concentration thresholds associated with short-term mucosal healing: 28.3 μg/mL (AUROC 0.64, 95% CI 0.53–0.75) at week 2, and 15.0 μg/mL (AUROC 0.69, 95% CI 0.59–0.79) at week 6 (11). *Post-hoc* analysis of ACT-1 and ACT-2 data in adults with UC demonstrated that achievement of serum IFX concentration threshold 22 μg/mL at week 6 was associated with clinical response at week 8 (17). In both our study and the aforementioned study by Papamichael et al. (11) the AUROC yields only borderline moderate diagnostic ability, possibly suggesting that IFX exposure is overall generally adequate by using an induction weight-based strategy with doses provided in frequent intervals. However, assembling a larger sample size of patients with variability in severity of disease, age, weight, dose, and timing may provide an improved understanding on how these variables contribute to IFX exposure and therapeutic response.

Limited data are available for comparison in children. There is a paucity of data on early induction IFX levels for children with UC. In a randomized, open-label multicenter study of children with moderate-to-severely active UC who received IFX induction, higher serum IFX concentrations at week 8 (≥41.1 μg/mL) were associated with higher proportions achieving efficacy endpoints (clinical response, 92.9%; mucosal healing, 92.9%; and clinical remission, 64.3%) compared to subjects with lower serum IFX concentrations (<18.1 μg/mL; 53.9, 53.9, and 30.8%, respectively) (8). A recent single center study of children with Crohn's disease by Clarkston et al. evaluated early IFX concentrations associated with achievement of target IFX levels (18). Among 72 children with Crohn's disease commencing IFX therapy (majority monotherapy), the pre-dose #3 IFX concentration ≥18 μg/ml was 82% sensitive, 82% specific (with 56% PPV, 94% NPV, AUROC 0.85, 95% CI 0.72–0.98) for achieving target levels >5 μg/ml at the start of maintenance (pre-dose #4). The authors then demonstrated that pre-dose #2 IFX concentration ≥29 μg/ml was the optimal cut-point to target pre-dose #3 ≥18 μg/ml (AUROC 0.82, 95% CI 0.70–0.94).

The currently approved induction regimen for IFX is 5 mg/kg at weeks 0, 2, and 6. However, concerns have risen for higher inflammatory burden and fecal IFX loss in active UC, resulting in lower serum IFX concentrations and therefore requiring an intensified schedule to achieve adequate concentrations. Seow et al. demonstrate that 56% of adults with UC had a low serum IFX concentration (median 2.1 μg/ml) at 4 weeks and undetectable serum IFX concentration at 6 weeks (10). Ungar et al. demonstrate lower IFX trough serum concentrations at 2 weeks (mean 7.15 ± 5.3 μg/mL) for acute severe UC compared to moderately severe UC (mean 14.4 ± 11.2 μg/mL) (19). Church et al. retrospectively evaluated an intensified induction (mean induction dose ≥7 mg/kg or interval between dose #1 and dose #3 ≤ 5 weeks) in steroid-refractory pediatric UC patients; an intensified induction was associated with higher chance of clinical remission (hazard ratio 3.2, *p* = 0.02) and lower chance of colectomy (hazard ratio 0.4, *p* = 0.05) compared to standard regimen (20). A systematic review by Hindryckx et al. identified uncontrolled studies that suggest a benefit for intensified induction for adults with acute severe UC, primarily in reducing early (3-month) colectomy rates (21). At the time of our study conduct, intensified IFX induction was intermittently performed. Nine subjects received dose #3 ≥ 7 mg/kg, 1 subject received dose #3 ≤ 35 days, and 3 received both [intensified IFX=13 subjects (38%)]. Neither interval shortening nor increased IFX dose were associated with reaching pre-dose #3 IFX threshold or week 8 clinical outcomes. The incremental benefit of IFX intensification on short and long term clinical, biomarker, and endoscopic outcomes requires further evaluation in a prospective study.

Evaluating factors impacting serum IFX concentrations, subjects <10 years and subjects with lower baseline weight had lower pre-dose #3 serum IFX concentrations. Adedokun et al. evaluated the pharmacokinetics of IFX in children with UC and demonstrated that median serum IFX concentrations at several time points during induction and maintenance were ~20% lower in children than adults (8). Using integrated data from pediatric patients with UC, Crohn's disease, juvenile rheumatoid arthritis, and Kawasaki disease, Xu et al. showed through population pharmacokinetic analysis that IFX clearance showed non-linear dependence on body weight and that systemic IFX exposure in very young children (aged 2 to 6 years) was predicted to be ~40% lower compared to adults (22). Therefore, the discrepancy between a linear dosing regimen of 5 mg/kg and a non-linear relationship between body weight and IFX clearance may predispose younger children to insufficient IFX exposure. This is affirmed by a recent study by Jongsma et al. which evaluated IFX trough levels in 110 young children (age <10 years) and 105 older children (age ≥ 10 years) (9). The authors showed that 72% of young children had inadequate IFX trough levels at the start of maintenance (<5.4 μg/mL) and young children required higher dose per 8 weeks compared to older children for the first year of IFX treatment [young children; 9.0 mg/kg (IQR 5.0–12.9) vs. older children; 5.5 mg/kg (IQR 5.0–9.3); *p* < 0.001] while maintaining similar overall duration of response to IFX. To affirm the uniqueness of pediatric pharmacokinetics, the median half-life in our study following IFX dose #1 in our study was 6.04 days (IQR 5.3–7.9 days) in comparison to reported median half life for IFX ~14 days (23). Further studies are required to explore optimal dosing regimen for children of young age and/or lower body weight to better define this patient population and ensure adequate IFX exposure to optimize response.

In our study, the majority of children achieved clinical remission at week 8 along with statistically significant improvement in serum biomarkers of inflammation. However, fecal calprotectin continued to remain elevated at week 6, likely suggesting that mucosal healing lags after clinical remission. This is consistent with results from a prospective multicenter cohort study of children with UC which demonstrated that PUCAI is more responsive to change in short time periods of re-evaluation than fecal calprotectin in severe UC (24). In a study of 36 children with Crohn's disease and UC, though 37% had normal fecal calprotectin <100 μg/g at 2 weeks following IFX start, only 8.3% had fecal calprotectin <100 μg/g at 6 weeks (25).

The strengths of our study center on the real world, multicenter, prospective nature of our study and the contribution of IFX trough levels during induction in pediatric UC to the very limited existing body of evidence. We acknowledge the limitations of our study. We selected a clinical outcome, PUCAI, as the endpoint of our study. Indeed, mucosal healing is a gold standard. However, in children, re-evaluation endoscopy is challenging as it is an invasive procedure often requiring anesthesia. Therefore, we selected a clinical outcome with strong validity to optimize feasibility; a PUCAI-defined remission has high concordance with complete mucosal healing at week 8 and PUCAI scores post-induction predict 1-year steroid-free remission (26). We recognize the importance of biomarker remission as a on outcome; however adherence to stool collection for fecal calprotectin was poor. Though this was included in our study protocol, only 7 subjects provided a complete series of three stool samples. Another limitation is the short duration of follow-up to 8 weeks. Further follow-up would enable improved understanding into how weight and age could impact achievement of medium and long-term serum IFX concentrations and clinical outcomes. Lastly, the size of our cohort limits the ability to make comparisons between groups.

In conclusion, in this multi-center prospective study of real-world clinical practice, the majority of children who undergo IFX induction for UC achieve short term clinical remission. IFX concentrations ≥33 μg/mL prior to the second dose are associated with week 8 clinical remission. As young age and low body weight impact IFX concentration, prospective studies with proactive adjustment in pediatric patients with UC should be carried out to optimize response to therapy. Clinicians caring for children with UC should adjust and monitor IFX dosing and interval to obtain therapeutic response.

## Data Availability Statement

The raw data supporting the conclusions of this article will be made available by the authors, without undue reservation.

## Ethics Statement

The studies involving human participants were reviewed and approved by Health Research Ethics Boards of University of Calgary, University of Alberta, University of British Columbia, and University of Manitoba. Written informed consent to participate in this study was provided by the participants' legal guardian/next of kin.

## Author Contributions

JD was involved in study concept and design, acquisition of data, analysis and interpretation of data, drafting of the manuscript, critical revision of the manuscript for important intellectual content, and final approval of submitted version. KJ, WE-M, EW, MC, IW, and HH were involved in study concept and design, acquisition of data, analysis and interpretation of data, critical revision of the manuscript for important intellectual content, and final approval of submitted version. CG was involved in analysis and interpretation of data, critical revision of the manuscript for important intellectual content, and final approval of submitted version. RP was involved in study concept and design, analysis and interpretation of data, critical revision of the manuscript for important intellectual content, and final approval of submitted version. All authors contributed to the article and approved the submitted version.

## Conflict of Interest

JD consultancy fees from Janssen, Abbvie, and Merck. Speakers bureau fees from Abbvie. WE-M consultancy fees from Janssen, Abbvie, and Merck. Speakers bureau fees from Abbvie. Financial support for research from Janssen. HH consultancy fees from Janssen, Abbvie and Merck. Financial support for research from Janssen, Takeda, Allergan. Education support from Janssen, Abbvie and Abbott. EW consultancy fees from Abbvie. Speaker for AbbVie, Janssen, Nestle. RP consultancy fees from AbbVie, Abbott, Alba Therapeutics, Allergan, Amgen, Aptalis, AstraZeneca, Atlantic Healthcare, Baxter, Biogen Idec, Boehringer Ingelheim, Bristol-Myers Squibb, Celgene, Cosmo Technologies, Coronado Biosciences, Cubist, Eisau Medical Research, Elan, Eli Lilly, enGene, EnteroMedics, Exagen Diagnostics, Ferring, Genentech, Genzyme, Gilead, Given Imaging, GSK, Hospira, Human Genome Sciences, Janssen, Merck & Co., Merck Research Laboratories, Merck Serono, Millennium, Nisshin Kyorin, Novo Nordisk, Pfizer Inc, Qu Biologics, Receptos, Relypsa, Salient, Salix Pharmaceuticals, Santarus, Shire, Sigmoid Pharma, and Takeda. Speakers bureau fees from AbbVie, Aptalis, Celgene, Ferring, Janssen, Merck, Pfizer Inc, Prometheus Laboratories, Shire, and Takeda. Financial support for research from AbbVie, Ferring, Janssen, Shire, and Takeda. The remaining authors declare that the research was conducted in the absence of any commercial or financial relationships that could be construed as a potential conflict of interest.

## Publisher's Note

All claims expressed in this article are solely those of the authors and do not necessarily represent those of their affiliated organizations, or those of the publisher, the editors and the reviewers. Any product that may be evaluated in this article, or claim that may be made by its manufacturer, is not guaranteed or endorsed by the publisher.

## Supplementary Material

Supplementary Figure 1 ROC analysis for infliximab serum concentrations at week 1, pre-dose #2, and pre-dose #3 stratifying subjects with and without clinical remission at week 8.

Supplementary Figure 2 Flow of subjects. *Includes one subject who proceeded to colectomy.

Supplementary Table 1 Test characteristics of pre-dose #2 and pre-dose #3 infliximab concentration cut-offs with week 8 clinical remission. IFX, infliximab; AUROC, area under receiver operator curve.

Supplementary Table 2 Univariate analysis. IFX, infliximab; CRP, c-reactive protein.

Supplementary Table 3 STROBE checklist for cohort studies.

## Abbreviations

BMI: body mass index
CRP: c-reactive protein
ESR: erythrocyte sedimentation rate
IFX: infliximab
IQR: interquartile range
PUCAI: pediatric ulcerative colitis activity index
ROC: receiver operating characteristics
UC: ulcerative colitis.
