# Supplementary figures and images for: Early Serum Infliximab Levels in Pediatric Ulcerative Colitis
Source: Front Pediatr. 2021 Jul 29;9:668978. doi: 10.3389/fped.2021.668978 (PMC8358797; doi:10.3389/fped.2021.668978)

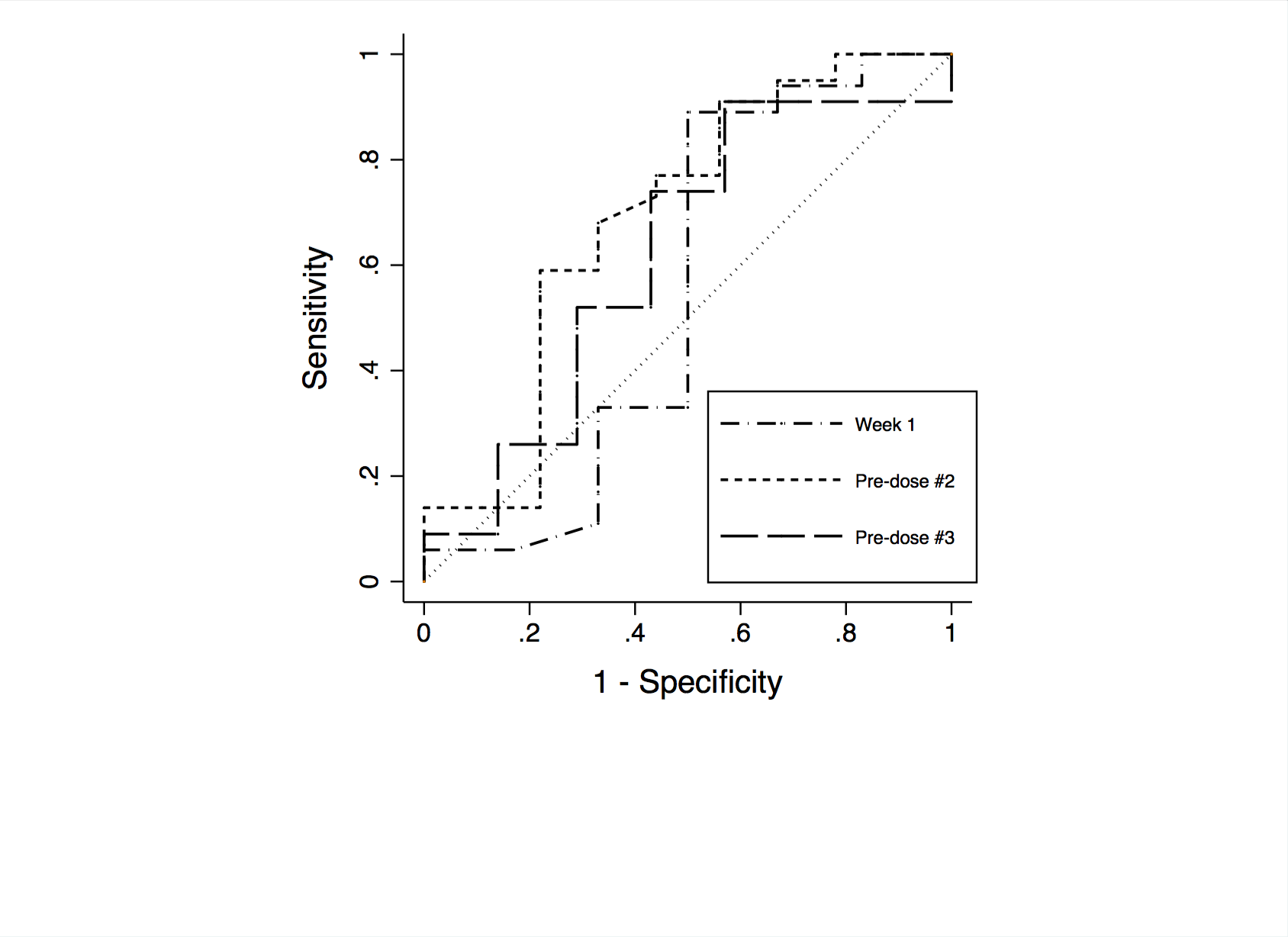

Supplement: Supplementary Figure 1 — ROC analysis for infliximab serum concentrations at week 1, pre-dose #2, and pre-dose #3 stratifying subjects with and without clinical remission at week 8. [file Image_1.TIF]
