# Supplementary material for: Early Serum Infliximab Levels in Pediatric Ulcerative Colitis
Source: Front Pediatr. 2021 Jul 29;9:668978. doi: 10.3389/fped.2021.668978 (PMC8358797; doi:10.3389/fped.2021.668978)
Supplement: Supplementary Figure 2 — Flow of subjects. *Includes one subject who proceeded to colectomy. [file Data_Sheet_1.DOCX]

*Includes one subject who proceeded to colectomy
