# Supplementary material for: Early Serum Infliximab Levels in Pediatric Ulcerative Colitis
Source: Front Pediatr. 2021 Jul 29;9:668978. doi: 10.3389/fped.2021.668978 (PMC8358797; doi:10.3389/fped.2021.668978)
Supplement: Supplementary Table 1 — Test characteristics of pre-dose #2 and pre-dose #3 infliximab concentration cut-offs with week 8 clinical remission. IFX, infliximab; AUROC, area under receiver operator curve. [file Table_1.docx]

**Supplemental Table 1.** Test characteristics of pre-dose #2 and pre-dose #3 infliximab concentration cut-offs with week 8 clinical remission

| **Infliximab concentration**  **(ug/mL)** | **Sensitivity**  **(%)** | **Specificity (%)** | **Positive predictive value**  **(%)** | **Negative predictive value**  **(%)** |
| --- | --- | --- | --- | --- |
| Pre-dose #2  ≥ 31  ≥ 32  ≥ 33  ≥ 34  ≥ 35 | 66.7  62.5  62.5  58.3  58.3 | 60.0  60.0  66.7  70.0  70.0 | 80.0  79.0  83.3  82.4  82.4 | 42.9  40.0  40.0  41.2  41.2 |
| Pre-dose #3  ≥ 12  ≥ 13  ≥ 14  ≥ 15  ≥ 16 | 91.7  87.5  87.5  87.5  83.3 | 30.0  30.0  30.0  30.0  30.0 | 75.9  75.0  75.0  75.0  74.07 | 60.0  50.0  50.0  50.0  50.0 |

IFX – infliximab, AUROC – area under receiver operator curve
