# Supplementary material for: Early Serum Infliximab Levels in Pediatric Ulcerative Colitis
Source: Front Pediatr. 2021 Jul 29;9:668978. doi: 10.3389/fped.2021.668978 (PMC8358797; doi:10.3389/fped.2021.668978)
Supplement: Supplementary Table 2 — Univariate analysis. IFX, infliximab; CRP, c-reactive protein. [file Table_2.docx]

**Supplemental Table 2.** Univariate analysis

|  | **Odds ratio for week 8 clinical remission**  **(95% confidence interval)** | **Odds ratio for**  **pre-dose #2 serum IFX concentration ≥ 33.0 μg/mL**  **(95% confidence interval)** |
| --- | --- | --- |
| **Male gender** | 0.90  (0.20-4.08) | 2.20  (0.50-9.61) |
| **Young age**  **(<10 years)** | 0.61  (0.12-3.27) | 0.40  (0.08-2.06) |
| **High baseline**  **CRP (>10.0 mg/L)** | 1.97  (0.41-9.52) | 0.35  (0.10-1.43) |
| **Low baseline albumin**  **(<33 g/L)** | 6.43  (0.70-59.17) | 0.58  (0.13-2.48) |
| **Body mass index** | 0.97  (0.90-1.05) | 1.15  (0.95-1.40) |
| **IFX dose #1 ≥ 7 mg/kg** | N/A | 3.20  (0.67-15.38) |
| **IFX dose #3 ≤ 35 days**  **from dose #1** | N/A | N/A |
| **Intensified IFX schedule** | 1.43  (0.29-7.12) | N/A |

IFX – infliximab, CRP – c-reactive protein
